# Toward the Decarbonization of Ammonia Production through the Gradual Incorporation of Green Hydrogen

**DOI:** 10.1021/acs.iecr.5c03851

**Published:** 2026-02-24

**Authors:** João Fortunato, Diogo A. C. Narciso, Henrique A. Matos

**Affiliations:** Centro de Recursos Naturais e Ambiente, Departamento de Engenharia Química, Instituto Superior Técnico, 37809 Universidade de Lisboa, Av. Rovisco Pais 1, 1049-001 Lisboa, Portugal

## Abstract

This work addresses the decarbonization of the ammonia industry, which relies almost exclusively on the Haber-Bosch (HB) process and accounts for more than 1% of anthropogenic carbon dioxide emissions. The first section of the HB process, the Steam Methane Reforming (SMR), is identified as the primary target for decarbonization, where fossil fuels are used as (i) feedstock for hydrogen (H_2_) production and (ii) a source for process heat. A methodology is proposed to gradually incorporate green H_2_ in the HB process, thus, reducing fossil fuel intake. The methane-fed HB process is modeled in *Aspen Plus*, where several process modifications are proposed. This includes an analysis of the most relevant point of green H_2_ injection and how to adapt plant operation to satisfy all process constraints, while minimizing methane consumption. The process limitations that are subject to this operation strategy were identified by increasing the green H_2_ incorporation fraction. The main bottleneck of this strategy relates to SMR operation, namely the increase in the secondary reformer’s outlet temperature. A partial bypass of the primary reformer is suggested to prevent this unit from overheating. This additional modification proved effective in controlling the temperature, enabling green H_2_ incorporation of up to 60% while satisfying all process constraints.

## Graphical abstract

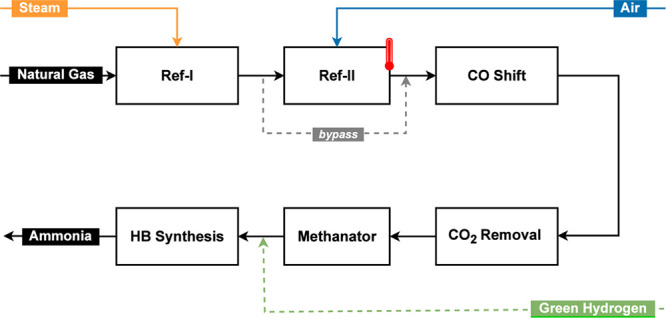

## 1 Introduction

Nitrogen (N_2_) is a key nutrient for plant growth and development and has been extensively used in the agricultural sector as a fertilizer to increase crop productivity and maintain soil fertility.^1^ Since the beginning of the 19th century, the nitrogen-based fertilizer market has relied on various sources of this nutrient (e.g., urea).

At the beginning of the 20th century, the agricultural sector was still pressured to find alternative N_2_ sources to address the increasing food demand posed by an exponentially growing population. In 1909, German chemist Fritz Haber successfully fixed atmospheric N_2_ by synthesizing ammonia (NH_3_) through its reaction with hydrogen (H_2_), using an iron-based catalyst:^2^

1 $$N_{2}+3H_{2}⇆2NH_{3}$$

In 1913, Carl Bosch scaled up the synthesis of NH_3_, and the process became known as the Haber-Bosch (HB) process. The contributions of both chemists were acknowledged with the Nobel Prize in Chemistry in 1918 and 1931, respectively.

NH_3_ became the world’s second-most-produced chemical compound, with around 183 million tons produced annually.^3^ Around 85% of NH_3_ production is used to produce nitrogen fertilizers, such as urea and ammonium nitrate.^3^ It is estimated that half of the world’s population directly depends on the food production enabled by applying these fertilizers.^1,4^ The remaining 15% of production is used in the pharmaceutical industry, textiles, explosives, and deNO_*x*_ technologies.^3^

The global scale of the HB process became so relevant that it is currently responsible for consuming 45% of the H_2_ produced, 2% of the final energy consumed, and 1 to 2% of the world’s carbon dioxide (CO_2_) emissions.^3,5^ This is justified by the significant reliance on fossil fuels, not only for heat generation but also primarily for H_2_ production.

Approximately 75% of NH_3_ is produced by consuming light hydrocarbons, such as natural gas and naphtha (gray NH_3_).^3^ This technology enables H_2_ production through Steam Methane Reforming (SMR) and is regarded as the Best Available Technology (BAT) due to its high efficiency, lower emissions, and reduced investment requirements.^6^ The remaining 25% is generated from coal and heavy fuel oil (HFO), which uses less efficient and more polluting processes (brown NH_3_).^3^ The NH_3_ produced through these methods is often referred to as first Generation NH_3_, with carbon footprints ranging from 1.6 (gray NH_3_) to 3.8 t CO_2_/t NH_3_ (brown NH_3_).^7,8^

The consumption of fossil fuels has come under increasing scrutiny due to its effect on rising Greenhouse Gas (GHG) levels in the Earth’s atmosphere. This is a growing international concern, and “decarbonization” initiatives are multiplying with a focus on carbon-neutral societies. The European Union (EU) has a series of policies in progress to promote the decarbonization of its industry, including the phasing out of free emission rights and the Renewable Energy Directive (RED) III.^9–11^ In this context, Carbon Capture and Storage (CCS) technologies are potential options for directly lowering the level of CO_2_ emissions from NH_3_ plants. However, implementing CCS does not address the reliance on fossil fuels, which is increasingly being challenged by EU policies focused on decarbonization and defossilization policies.^10,11^

These ambitious goals have prompted heightened efforts to transition NH_3_ production from the energy-intensive and high-carbon-featuring HB process. New approaches to manufacturing NH_3_ have been researched in various fields, among which electrochemical, electrocatalytic, photocatalytic, and photoelectrocatalytic synthesis can be identified as emerging routes.^9^

Although these emerging routes are promising for decarbonizing the NH_3_ production process, low Technology Readiness Levels (TRL1–3) are still reported. Their application still depends, among other factors, on the development of new catalysts and new electrolyte components that enable optimization of conversion, efficiency, and selectivity.^9,12,13^ They are not expected to provide an industrially viable route in the short term.^13^ In the future, this third generation of NH_3_ manufacturing processes is expected to enable a fully green path for its production.^7^

The most advanced technology available, with TRL 8–9, is the electrification of the HB process, which involves producing H_2_ through water electrolysis to replace fossil feedstock consumption and obtaining N_2_ through air separation.^2^ Supplying the entire process with renewable energy (RE) makes it possible to produce low-carbon or green NH_3_. The challenge with this technology is how the process can manage the variability of RE sources such as wind and solar energy. Some of the equipment, namely, electrolyzers, is quite flexible and can handle the variability of the RE power. Without further options, this means supplying HB synthesis with a variable H_2_ and/or N_2_ flow rate. As a highly integrated process, the HB synthesis is inherently sensitive to unsteady flow rates, which may cause fluctuations in the catalyst temperature and potentially impact its performance or lifespan. Maintaining a stable supply of H_2_ and N_2_ is therefore essential to prevent off-design operation and guarantee optimal NH_3_ synthesis, which can be achieved through effective RE management. Numerous studies are available in the literature on the design and operation of green NH_3_ systems, going beyond the traditional chemical process and considering also RE production and the operation of auxiliary units such as batteries.^14–19^ An extensive review of this type of study is provided in the work of Narciso et al.^9^

Although such dynamic operation poses significant challenges, extensive research and industrial development have been dedicated to understanding and enhancing the flexibility of the HB synthesis. Major technology licensors have developed adaptable HB synthesis loops that can handle variations in feed composition and flow rates without compromising performance. Recent academic research has further explored the dynamic behavior of these systems, examining strategies such as advanced process control,^20^ optimization,^21^ and three-dimensional computational fluid dynamics simulations^22^ to predict and manage transient responses.^23,24^ These combined efforts show that, despite the challenges, flexible HB synthesis operation is possible and that process design and control strategies can successfully reduce risks linked to variable RE inputs.

Although green NH_3_ projects are progressing worldwide, many are still in the feasibility stage, as green H_2_ costs remain significantly higher than gray H_2_, making fully green NH_3_ economically unviable in the near term.^25,26^

An immediate transition to fully green NH_3_ would also mean a significant loss of competitiveness for the current NH_3_ industry due to the depreciation of substantial industrial assets resulting from costly past investments. To meet the EU’s targets and ensure their competitiveness in the global NH_3_ market, some European NH_3_ producers have tried to partially decarbonize their production by incorporating green H_2_ into their conventional plants.^27^ In the fertilizer industry, this partial decarbonization is especially important because part of the CO_2_ generated during the NH_3_ process is used as a feedstock for products like urea. By contrast, total decarbonization of NH_3_ production could also be key to unlocking more promising uses for this molecule: NH_3_ is often proposed as a carbon-free fuel for maritime transportation or as an H_2_ carrier.^5,7^ For these new applications, decarbonizing NH_3_ production is even more important, as its use is expected to help achieve the decarbonization targets established by these markets.^3^

The retrofitting of NH_3_ plants is discussed in the study by Musa et al.,^28^ which proposes replacing the conventional SMR with a novel dual-reactor reforming technology, without considering the integration of green H_2_. In contrast, the study by Pan et al.^29^ addresses the use of green H_2_; however, it assumes a complete replacement of H_2_ produced via coal gasification with green H_2_, rather than a gradual or partial retrofit of existing plants. The first study on the retrofitting of a conventional NH_3_ plant by the gradual incorporation of green H_2_ is presented in the study of Isella et al.^30^ Here, a plant with a daily production capacity of 2000 tNH_3_ is considered and modeled via the UniSim Design software. The authors identified some key challenges derived from integrating green H_2_, namely, the need to ensure the minimum operating capacities (due to the reduction of natural gas) and the overheating of some equipment. By defining a lower bound of 80% of the nominal capacity for the volume throughput and +100 °C as a maximum allowed temperature increase, the authors achieved a maximum of 20% process decarbonization from incorporating 22% green H_2_.

To the best of the authors’ knowledge, no article has been published since then addressing the retrofitting of conventional NH_3_ plants via the incorporation of green H_2_.^30^ This work presents a new contribution in this field, namely, addressing the following objectives:

- Understand the main effects of increasing the green H_2_ incorporation fraction in the conventional NH_3_ plant, specifically the impacts reported in SMR units.
- Propose a solution to mitigate those impacts and enable a high incorporation of green H_2_ in conventional NH_3_ plants. This includes adding new process streams, connecting the existing process units, and adjusting operating conditions.
- Identify open research questions and future work.

## 2 Methodology

To study the decarbonization of ammonia (NH_3_) production, a conventional natural gas-based plant was simulated in *Aspen Plus V14*. The plant has a daily capacity of 670 ton of NH_3_ and can be divided into two sections: the SMR section and the HB Synthesis section, as illustrated in Figure 1. The SMR section was modeled using the local example in *Aspen Plus V14* (under the *Fertilizers* folder – file “Ammonia”), where several modifications were made to (i) reach the target capacity, (ii) allow green hydrogen (H_2_) incorporation, and (iii) align the model with literature and industrial data.^31^ While the plant features both the SMR and HB synthesis sections, this work focuses primarily on the SMR and explores how syngas production can be decarbonized through the gradual incorporation of green H_2_. As a result, the HB synthesis section is depicted more simply without additional details. The overall flowsheet of the SMR section and the detailed flowsheets for the main subsections (desulfurization, reforming, CO Shift, CO_2_ removal, and methanation) are provided in Figures S1–S6 of the Supporting Information. Additionally, the key data for these main units can be found in Tables S1–S4.

**Figure 1:**
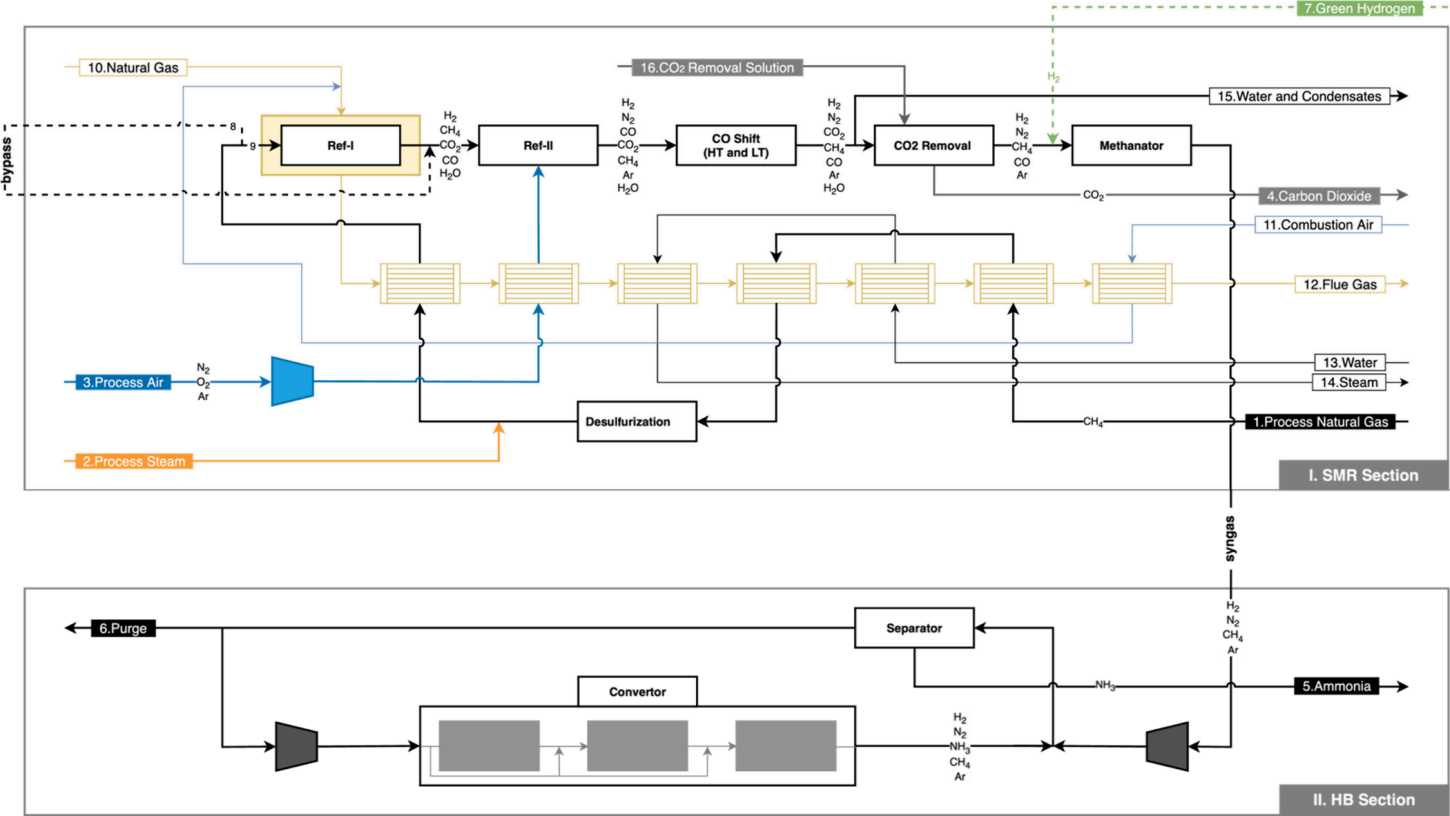
Diagram of the conventional NH_3_ production process: SMR section and HB section, upper and lower boxes, respectively (solid lines represent the flowsheet of the conventional HB plant, while dashed lines represent the modifications introduced in the present work).

In Figure 1, the conventional HB process is shown. It includes all units within the gray boxes, while the proposed process modifications are depicted as dashed lines crossing these gray boxes. The modifications include a new stream for the supply of green H_2_ from an external source (stream 7) and a new bypass stream between the Primary and Secondary Reformers (ref-I and ref-II, respectively). The proposed modifications were considered to improve the operational flexibility of the conventional NH_3_ plant and are examined in the following sections.

### 2.1 SMR Section

Natural gas, steam, and air are the primary feedstocks in the SMR section, where H_2_ is produced to deliver a stream rich in H_2_ and nitrogen (N_2_) in a 3:1 molar ratio (referred to as *syngas*). To this end, the SMR section comprises multiple units, which are briefly described next.

Natural gas (stream 1 in Figure 1) is initially purified through hydrodesulfurization to prevent catalyst poisoning in multiple process units. The natural gas stream is then heated, mixed with steam (stream 2), and fed into a reforming unit. This unit is the core of the SMR section, crucial for gray H_2_ production, and is divided into two reactors: primary and secondary reformers.

In the primary reformer (Ref-I), H_2_ is produced by steam consumption, as shown in eqs 2 and 3. Steam is supplied in excess to minimize carbon formation reactions and shift the equilibrium toward the products.

2 $$CH_{4}+H_{2}O⇆CO+3H_{2}$$

3 $$CO+H_{2}O⇆CO_{2}+H_{2}$$

The effluent gas mixture from ref-I is mixed with compressed hot process air (stream 3) and fed into the secondary reformer (Ref-II), where the conversion of unreacted methane (CH_4_) is completed. The conversion is accomplished through CH_4_ partial oxidation, as shown in eq 4, and total oxidation, as indicated in eq 5.

4 $$CH_{4}+0.5O_{2}⇆CO+2H_{2}$$

5 $$CH_{4}+2O_{2}⇆CO_{2}+2H_{2}O$$

However, the importance of this stage extends beyond H_2_ production. The air feed supplied to ref-II provides the N_2_ needed for NH_3_ synthesis, as shown in eq 1. Since all N_2_ used for NH_3_ synthesis must come from this air intake, its flow rate is limited by the necessity to maintain the overall H_2_/N_2_ ratio at the entrance of the HB section. Consequently, the operation of the entire SMR, especially ref-II, is directly restricted by the N_2_ required for NH_3_ synthesis. In addition, argon (Ar) is also introduced with the airflow and remains chemically inert in all of the process units.

The gas mixture exits the reforming section and is cooled, and the carbon monoxide (CO) in this stream is shifted into CO_2_, as shown in eq 3. The reaction consumes part of the excess steam introduced in ref-I and produces additional H_2_. This step is divided into two stages to improve the conversion: a high-temperature stage (HT, 350–380 °C) and a low-temperature stage (LT, 200–220 °C). H_2_ production concludes in this unit, and the subsequent process steps are necessary only to ensure the quality of the synthesis gas.

Carbon dioxide (stream 4) is then removed by chemical or physical absorption processes, commonly through the use of amine solutions or hot potassium carbonate. The choice of the CO_2_ removal process may depend on the desired purity of this compound, which can be utilized by the carbonated beverage industry or integrated into urea production.

The final step in the SMR process is the methanation of carbon oxides, as shown in eqs 6 and 7. This enables the removal of oxygen compounds from the gas mixture that would act as catalysts’ poisons in the HB synthesis section.^31^ Carbon oxides are converted to water, which is then removed from the system by using molecular sieve adsorbers.

6 $$CO+3H_{2}⇆CH_{4}+H_{2}O$$

7 $$CO_{2}+4H_{2}⇆CH_{4}+2H_{2}O$$

This step ensures strict industrial requirements for preventing oxygen compounds are satisfied, usually keeping the oxygen equivalent in the HB section feed below 10 ppm.^32^ The gas mixture obtained at the end of the SMR section is mainly composed of H_2_ and N_2_ in a 3:1 molar ratio, with traces of CH_4_ and Ar, which are chemically inert in the HB Synthesis section.

The description above concerns the main process line of the SMR section, which deals directly with the gas mixture’s synthesis. The SMR section also includes an auxiliary process line designed to meet the temperature requirements of the main process line, as depicted in Figure 1. This line is divided into the radiant and the convection stages.

The radiant stage consists of a fired box in which natural gas (stream 10) is combusted with air (stream 11). Inside the fired box, tubes filled with catalyst are crossed by the initial mixture of desulfurized natural gas and steam (ref-I). The combustion supplies the energy necessary for the endothermic steam reforming reaction, as shown in eq 2, through radiation.

The flue gas effluent from the radiant stage passes through a series of heat exchangers where energy is transferred through convectional heat transfer. The convection stage reduces the flue gas’s thermal energy while simultaneously preheating the process and combustion air and natural gas and (optionally) generating steam. At the end of the convection stage, the flue gas is released to the atmosphere (stream 12).

A summary of the reactor models and reaction specifications used in the model of this section is presented in Table 1. Further details can be found in the Supporting Information.

**Table 1:** Reactor Models and Reaction Specifications in *Aspen Plus* for the Main Units Are Shown in the SMR Section

unit	reactor model	reaction specification
Desulfurization	*RStoic*	Fractional Conversion
Primary Reformer	*RPlug*	Kinetic Model
Secondary Reformer	*RGibbs*	Chemical Equilibrium
CO Shift - HT	*RPlug*	Kinetic Model
CO Shift - LT	*RPlug*	Kinetic Model
Methanation	*RPlug*	Kinetic Model

### 2.2 Key Process Modifications

To achieve the desired capacity, the flow rates of the raw materials reported in the original model were adjusted along with the volume of the equipment, particularly the reactors specified by kinetic models. These adjustments considered the information and data available in the literature to model a more realistic process. This was especially important for modeling the Secondary Reformer, as the original Aspen Plus model only considers a different chemical reaction in this unit, eq 8, cannibalizing some of the H_2_ produced in Primary Reformer.

8 $$H_{2}+0.5O_{2}⇆H_{2}O$$

In this work, the introduction of green H_2_ was considered immediately upstream of the methanation step (SMR section). If high-grade H_2_ is available, one might consider introducing it directly into the synthesis loop, as proposed by Isella et al.^30^ However, green H_2_ is commonly manufactured via water electrolysis, which typically includes traces of water and oxygen. To prevent catalyst poisoning, feeding green H_2_ upstream of the methanation unit is deemed to be the best choice to favor the removal of these molecules from the syngas stream.

The supply of green H_2_ was considered (stream *7*) without modeling the upstream electrolysis process. The concept of green H_2_, widely applied throughout this work, could therefore be extended to encompass all types of low-carbon H_2_, as the source of energy consumed to produce H_2_ was not assessed. This includes energy sources, such as solar, wind, or nuclear power. For solar and wind, which may have variability issues, maintaining a steady H_2_ supply as considered here might require energy storage solutions like buffer H_2_ storage sufficient for several days of operation. However, if not considered, particularly for small green H_2_ flow rates, the plant should retain the flexibility to operate temporarily in full conventional mode.

The fraction of green H_2_ supplied is defined in this work as an input parameter, which may be set between 0% (mimics of a conventional NH_3_ plant) and 100% (equivalent to a fully green NH_3_ plant, which in this extreme case would render the SMR process at least partly redundant). The Green Hydrogen Incorporation (GHI) fraction is defined in eq 9, where *Green H*_*2*_ represents the external supply of H_2_ (stream 7) and *Gray H*_*2*_ represents the H_2_ produced by SMR process units (both expressed as molar flow rates).

$$GHI\;\left[\%\right]=\frac{Green\;H_{2}\;Flowrate}{Green\;H_{2}\;Flowrate+Gray\;H_{2}\;Flowrate}=\frac{Green\;H_{2}\;Flowrate}{H_{2}\;Flowrate\;Syngas\;Stream}$$ 9

A bypass that diverts part of the unreacted CH_4_ and steam mixture from the primary reformer directly to the secondary reformer is also considered. A careful selection of this bypass fraction enables a more effective management of the performance of the reforming units, which in turn plays a vital role in enabling the proposed concept for high GHI. This fraction is defined in eq 10, where *Bypass Flow rate* and *Reformer I Inlet Flow rate* represent the respective molar flow rates of streams 8 and 9, respectively, immediately after the ref-I bypass split. In a conventional HB process, the Bypass Fraction is 0%. More broadly, and for any GHI, this variable takes the role of a process degree of freedom, which may be independently set to achieve the best performance. This is a key process modification and a significant novelty concerning prior work in the field.

10 $$\mathrm{Bypass Fraction}\;\left[\%\right]=\frac{Bypass\;Flowrate}{Bypass\;Flowrate+Reformer\;I\;Inlet\;Flowrate}$$

### 2.3 Methodology for Green Hydrogen Incorporation

#### 2.3.1 Process Operation Principles

Two operational strategies (S–I and S–II) were developed in this work to manage the GHI in NH_3_ plants. Their underlying principles are listed below:

- **S–I:** decreases the production of gray H_2_ by reducing natural gas and process steam intake in the SMR section. As GHI increases, this adjustment keeps the overall available H_2_ constant. N_2_ feed is kept constant, and so is the production of NH_3_. Air consumption may be slightly affected to compensate for the reduction of N_2_ intake in the natural gas stream (impurity).
- **S–II:** the gray H_2_ production remains unchanged, meaning that increasing the green H_2_ fraction increases the overall available H_2_. In this case, the inlet flow rate of process air must be increased, allowing for higher NH_3_ production.

Table 2 summarizes the main changes in the process according to the operating strategy considered.

**Table 2:** Effect on the Amount of Natural Gas, Steam Consumed, Air, and on the Amount of NH_3_ Produced with the Incorporation of Green H_2_, for Strategies S–I and S–II

	Natural Gas 1	Steam 2	Air 3	NH_3_ Prod. 6
**S–I**	↑	↑	≈	=
**S–II**	=	=	↑	↑

Both strategies lead to a certain degree of decarbonization of the process. The authors decided to follow S–I to carry out the present work, as it better represents the industrial reality of achieving a predefined NH_3_ target production and replacing gray H_2_ with green H_2_.

Note that in line with Table 2, S–I delivers a constant flow rate of H_2_ and N_2_ in the syngas stream transferred between the SMR and HB synthesis sections. The two chemical species constitute the main bulk of the syngas stream (∼99%mol), regardless of the extent of GHI in S–I. The main impact of this strategy is on the composition of inert species (CH_4_ and Ar) in the syngas mixture, which change very slightly with GHI, and thus have a minimal impact on the HB synthesis section. For this reason, this research work is focused on the operation of the SMR process in the context mentioned above.

#### 2.3.2 Process Operation Strategy

To achieve the high-level production objectives enumerated in Table 2, S–I was developed in detail and seeks the adjustment of the available process degrees of freedom to deliver the most efficient operation for all GHI scenarios. Consistent with the process flowchart in Figure 1, all process variables that can be adjusted are listed in Table 3. This table mainly lists flow rates for most variables, but it also includes the temperature of the flue gas (Flue Gas Out ref-I), which allows for more flexible interactions between the heat supplied in ref-I (radiant) and the various heat exchangers (convection section).

**Table 3:** Degrees of Freedom Considered for Green H_2_ Incorporation in the Modified NH_3_ Plant Model^a^

Degree of Freedom	Variable Type	Lower Bound	Upper Bound	Impact
Process Natural Gas (stream 1)	Flow rate [kg/h]	0	2 × 10^5^	Regulate gray H_2_ production
Process Steam (stream 2)	Flow rate [kg/h]	0	3 × 10^5^	Regulate gray H_2_ production
Process Air (stream 3)	Flow rate [kmol/h]	0	3 × 10^4^	Ensure the correct amount of N_2_
CO_2_ Removal Solution (stream 16)	Flow rate [kg/h]	0	1 × 10^6^	Ensure successful removal of CO_2_
Fuel Natural Gas (stream 10)	Flow rate [kg/h]	0	15 × 10^3^	Meet ref-I energy demand
Combustion Air (stream 11)	Flow rate [kg/h]	0	2 × 10^6^	Meet ref-I energy demand
Flue Gas Out ref-I	Temperature [°C]	850	980	Allows flexibility between radiant and convection sections
**ref-I Bypass**	**Fraction** [-]	**0**	**1**	**Avoids ref-II overheating**

a The boldface variable is only considered in the adapted SMR, section 3.2.

The SMR process is also subject to a set of constraints to ensure that the process operates within the desired ranges for operational efficiency and safety. Based on the literature and insights from SMR process engineers, a set of constraints has been compiled and listed in Table 4. Since the fired box and ref-I are modeled independently, a constraint is set to ensure the heat generated in the fired box, Q (Fired Box) < 0, sufficiently meets the energy supply in ref-I, Q (ref-I) > 0.

**Table 4:** Constraints Considered for Green H_2_ in the Modified NH_3_ Plant Model^a^

constraint	constraint type	lower bound	upper bound
Steam to Carbon Ratio in ref-I	Fraction [kmol/kmol]	2.9	2.9
Total H_2_ Production	Flow rate [kmol/h]	3000	3000
CO_2_ Content at the Outlet of the Removal Section	Molar Fraction [%mol]	0	0.01
Q (Fired Box) + Q (ref-I)	Heat Duty [MW]	-inf	0
Combustion Air to Fuel	Ratio [ton/ton]	20	20
H_2_ to N_2_ at Syngas	Ratio [kmol/kmol]	3	3
**ref-II Outlet Stream**	**Temperature** [°C]	**0**	**918**

a The boldface constraint is only considered in the adapted SMR, section 3.2.

Based on the information above, an optimization problem can now be formulated: it takes all of the variables listed in Table 3 as the optimization variables and the constraints in Table 4, as well as the GHI input imposed for different fractions. From a conceptual perspective, it suffices to define the optimization problem’s objective function, which is defined in eq 11.

11 $$\min imize\;f_{obj}=\mathrm{Pr}ocess\;Natural\;Gas\;Flowrate+Fuel\;Natural\;Gas\;Flowrate$$

Although economic parameters are not explicitly considered in this optimization, natural gas represents up to 70% of total production costs, which validates eq 11 as a meaningful objective function for initial screening studies.^33^ Furthermore, the definition of this objective function is fully aligned with the defined process decarbonization goals. It is important to note that in the current formulation, the costs of H_2_ are not directly considered, since the problem is viewed from the perspective of the production engineer, who must operate with a specified fraction of green H_2_, regardless of its cost. From a broader perspective, one of the key goals of this work is to demonstrate the technical feasibility of the proposed concept. From a strategic perspective, the formulation of the objective function to encompass all relevant factors, including the green H_2_ cost as a primary factor in the operation of retrofitted HB processes, will be the subject of future work.

To implement and solve the optimization problem defined above, the optimization module in *Aspen Plus V14* was used. The optimization variables were listed in the *Vary* tab, the constraints were specified in the *Objective & Constraints* tab, and the objective function was set in the *Define* tab.

## 3 Results and Discussion

### 3.1 Conventional SMR Operation

The main effects of incorporating green hydrogen (H_2_) and adapting the SMR operation via S–I were studied for fractions ranging from 0% (base case) to 10%. For this study, ref-I Bypass was not considered (*ref-I Bypass Fraction = 0*), nor the constraint on ref-II Outlet Stream (only the design variables and constraints not marked in bold in Table 3 and Table 4, respectively). To analyze the impact of GHI on system performance, the following metrics are defined

$$CH_{4}\;Savings\;\left[\%\right]=\frac{CH_{4}\;Flowrate\;\left(GHI_{0\%}\right)-CH_{4}\;Flowrate\;\left(GHI_{x}\right)}{CH_{4}\;Flowrate\;\left(GHI_{0\%}\right)}$$ 12

13 $$Total\;Decarbonization\;\left[\%\right]==\frac{\left[CO_{2,proc}+CO_{2,fuel}\right]Flowrate\;\left(GHI_{0\%}\right)-\left[CO_{2,proc}+CO_{2,fuel}\right]Flowrate\left(GHI_{x}\right)}{\left[CO_{2,proc}+CO_{2,fuel}\right]Flowrate\left(GHI_{0\%}\right)}$$

where *CH*_*4*_ accounts for the total Process and Fuel Natural Gas consumption (streams 1 and 10), *CO*_*2,proc*_ accounts for the CO_2_ flow rate removed in the CO_2_ Removal unit (stream 4), and *CO*_*2,fuel*_ accounts for the CO_2_ flow rate produced by fuel combustion (stream 12).

Although both metrics are inherently connected, they can yield different values for the same GHI fraction since carbon can exit the process as CH_4_, CO_2_, and CO in various streams that are not included in the definition of Total Decarbonization. For instance, although the savings of CH_4_ burned as fuel (stream 10) are linearly related to CO_2_ emissions (stream 12) via reaction stoichiometry (eq 5), this linear behavior does not apply in the process line with the consumption of Process Natural Gas (stream 1). The most notable example of this is the amount of CH_4_ that leaves the SMR section as an inert chemical in the syngas stream.

The two metrics match at the base case, as illustrated in Figure 2, but as the GHI increases, the flow rates of CO_2_ and CH_4_ in the process do not vary in the same proportion as a result of the operation of both ref-I and ref-II. In fact, as green H_2_ is increasingly introduced, the amount of carbon exiting the process as CH_4_ in the syngas stream decreases, which explains the difference between the two metrics defined. Eventually, as inert CH_4_ is purged in the HB Section alongside other compounds and potentially combusted as a fuel source, the associated CO_2_ emissions would offset and ultimately neutralize the discrepancies between these metrics. An illustration of the application of these metrics is presented in Table S7 (Supporting Information), where the carbon mole balances for the base case (GHI = 0%) and for GHI = 6% are examined.

**Figure 2:**
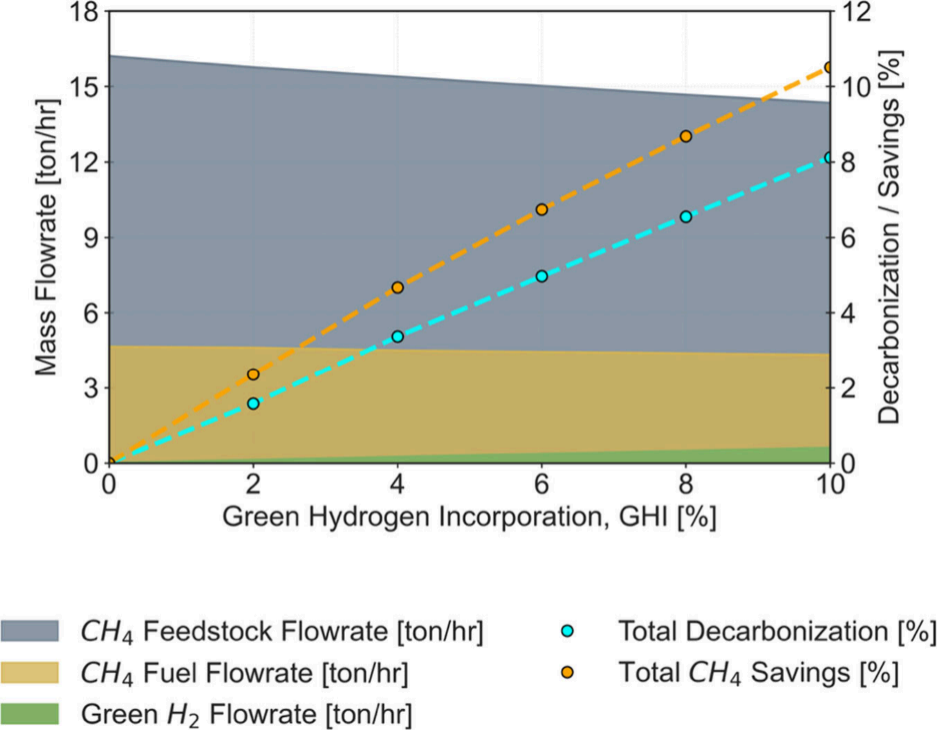
Natural gas and Green H_2_ flow rates (left axis), total CH_4_ savings, and decarbonization extent (right axis) for GHIs from 0 to 10%.

Figure 2 illustrates the optimized natural gas consumption, which is quantitatively expressed in mass flow rates (for process and fuel natural gas) and in terms of savings achieved compared with the base-case consumption values. The results are available in Table S5 of the Supporting Information.

The most significant savings in natural gas are noted in the main process line (around 11% for a GHI of 10%), primarily to reduce the production of gray H_2_ in the reforming units and accommodate the intake of green H_2_. Lower savings (around 8% for the same GHI of 10%) are reported in the auxiliary process line, indicating that less natural gas is needed to meet the energy demand for the endothermic reactions at ref-I. Reporting these savings is crucial from a scientific and technical perspective, as it helps quantify the potential economic benefits and track the ongoing decarbonization efforts within the process, providing a representation of the transition from a fully methane-based (100% gray) to a potential fully renewable (100% green) system. Additionally, it supports the identification of critical operation conditions and the assessment of mitigation strategies.

These results were expected, reflecting the effect of replacing gray with green H_2_. Still, they are also reflected in a decrease in the flow rate (or load) circulating through the reforming units. Figure 3 illustrates the mass flow reduction in both ref-I and ref-II, indicating a more significant decrease in the former. This is justified by the constant Process Air intake in ref-II, despite the reduction of both feedstocks in ref-I (Process Natural Gas and Process Steam). The nonlinear mass flow reduction observed in both reformers is a result of the material and energy balances affected by the green H_2_ incorporation, affecting the nonlinear kinetic and equilibrium behavior reported in ref-I and ref-II.

**Figure 3:**
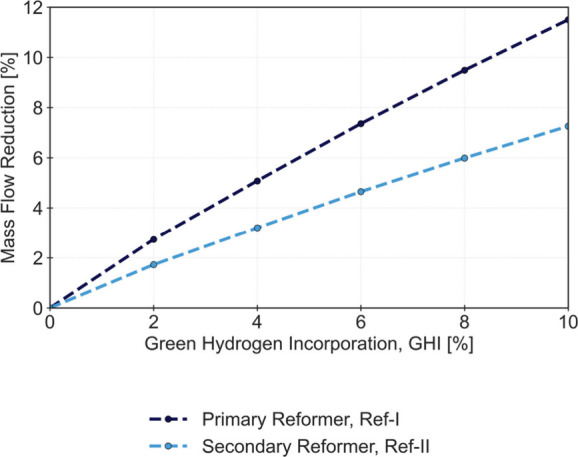
Mass flow rate reduction for ref-I and ref-II for an incorporation of Green H_2_ (GHI) of 0 to 10%.

The reduction in ref-I load results in longer residence times, which leads to a higher CH_4_ conversion and an increase in outlet temperature reported in this unit. The reduction of CH_4_ that circulates within the reforming units also contributes to a temperature increase in the outlet of ref-II. Since the amount of air remains roughly unchanged, the amount of oxygen consumed in ref-II also remains constant, which results in a lower CH_4_ to O_2_ ratio reported at the inlet of that unit. This reduction in the ratio favors the extension of the reaction represented by eq 5 compared to that represented by eq 4. Although the conversion of CH_4_ reported in this study increases with the GHI fraction, the distribution between the reactions results in a relatively decreased H_2_ production and increased water formation. Nonetheless, even as the GHI fraction rises, both reactions consume all the oxygen supplied by the atmospheric air intake. The higher exothermicity of the reaction represented by eq 5 justifies the overheating of the outlet stream as the fraction of green H_2_ is increased. Even with an incorporation of 10%, a temperature increase of over 80 °C is reported. The high temperatures achieved in this operational setting limit the extent of GHI in the SMR process, especially considering that process equipment materials were not designed for these harsh conditions. The results obtained are listed in Figure 4.

**Figure 4:**
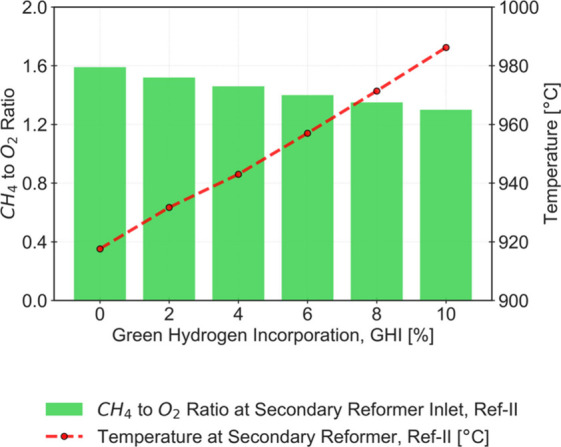
CH_4_ to O_2_ ratio at the ref-II inlet stream (left axis) and outlet ref-II temperature (right axis) for an incorporation of Green H_2_ (GHI) of 0 to 10%.

These results highlight two important effects that need to be considered when incorporating green H_2_ into a conventional ammonia (NH_3_) plant:

- The reduction of the load on the reforming equipment is particularly noticeable in ref-I (without any further modifications). Despite the reduced load processed by this equipment, proper operation can be maintained. However, a typical reformer has a specified minimum load at which burner-related issues begin to arise. Some reformer units can reduce their load to around 60% of their nominal capacity, although an even sharper reduction can be achieved with additional adjustments, such as increasing the steam-to-carbon ratio. This information is based on confidential industrial data provided to the authors.
- The outlet stream of ref-II is overheating. Considering the already high temperatures of the base case without green H_2_ incorporation (*Temperature Out ref-II* = 918 °C), it is uncertain whether a temperature increase can be supported, primarily due to metallurgical limitations. Applying the same criteria of Isella et al, which restrict the temperature rise of this unit by +100 °C,^30^ it is unlikely that the H_2_ fraction in the green H_2_ output could be significantly increased beyond approximately 10% without additional process adaptations. To overcome the overheating reported in ref-II, one may consider precooling the ref-II feed, resulting in a lower outlet temperature. However, this modification would require the addition of a dedicated heat exchanger, which lies outside the scope of the process integration modifications considered in this work. Moreover, a low feed temperature would favor total oxidation of CH_4_ (eq 5) over its partial oxidation (eq 4). The reduced H_2_ production (only achieved through partial oxidation) would need to be compensated by increased natural gas consumption.

In Section 3.2, an adapted SMR process is proposed, where the bypass fraction presented in Section 2.3 becomes an operational degree of freedom to safely achieve higher GHIs, while mitigating the effects highlighted above.

### 3.2 Adapted SMR Operation

The challenge of maintaining NH_3_ production while preventing overheating of ref-II and keeping the CH_4_ to O_2_ ratio can be addressed by increasing the amount of CH_4_ available in the ref-II inlet stream. By avoiding the processing of a portion of the CH_4_ in ref-I, it is possible to maintain the ratio unchanged. This diversion can be seen as a ref-I *bypass*, as suggested in Figure 1. However, this strategy incurs the cost of losing part of the H_2_ production from steam that is achieved only in ref-I, as shown in eqs 2 and 3.

Given this new adapted SMR, the same optimization problem was solved, but now defining the ref-I bypass fraction as an optimization variable (0 < *ref-I Bypass Fraction < 1*) for a more efficient process operation management, namely to prevent any overheating reported at the outlet stream of ref-II. The temperature constraint on ref-II Outlet Stream (last row of Table 4) is also enabled in this case.

The savings in overall natural gas consumption achieved by this new configuration are slightly lower, as shown in Figure 5. The results are available in Table S6 of the Supporting Information. A significant improvement is reported in the fuel burned in ref-I, which is justified by the lower energy demand in steam reforming reactions. The lower savings obtained for the process natural gas suggest a higher flow rate of CH_4_ is needed to produce the same gray H_2_ flow rate, compensating for the losses of the H_2_ produced through steam at ref-I. The extent of decarbonization achieved is similar, primarily due to the reduction in natural gas consumption as fuel at ref-I. Figure 5 also shows the fractions of the ref-I bypass required to maintain the outlet temperature of ref-II at 918 °C.

**Figure 5:**
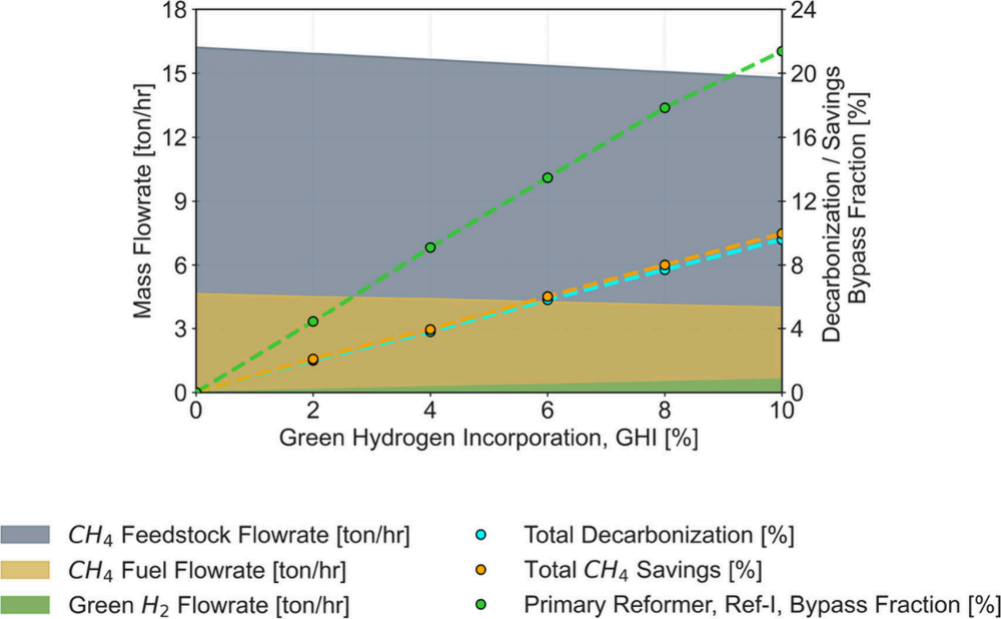
Natural gas and Green H_2_ flow rates (left axis), total CH_4_ savings, decarbonization extent, and ref-I bypass fractions (right axis) for an incorporation of Green H_2_ (GHI) of 0 to 10%.

The ref-I bypass fractions obtained are significant, and by examination of the mass flow reductions reported at ref-I and ref-II (Figure 6), a significantly sharper reduction is observed for ref-I, justified by the bypass operation.

**Figure 6:**
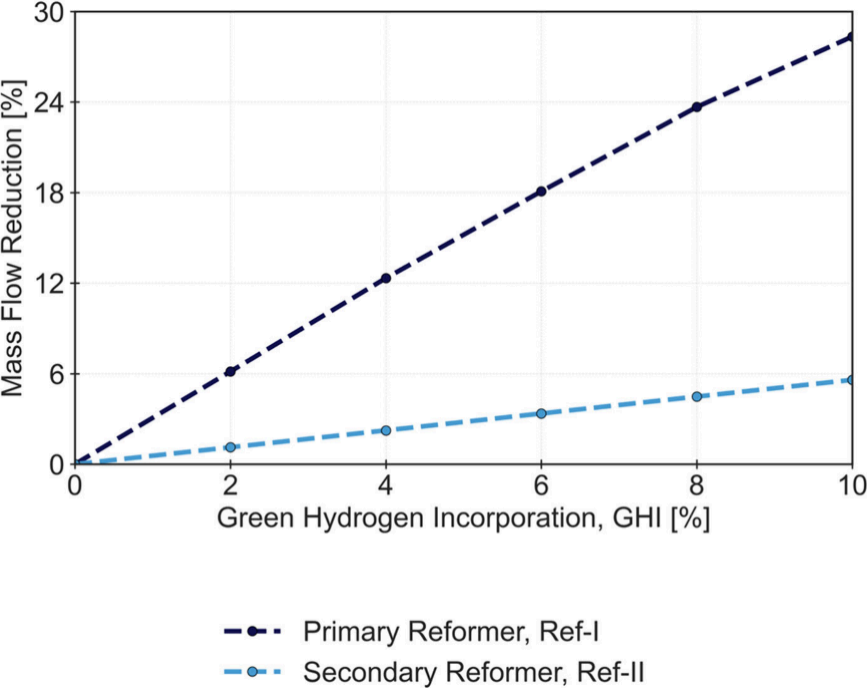
Mass flow rate reduction for ref-I and ref-II for an incorporation of Green H_2_ (GHI) of 0 to 10%, considering an adapted SMR.

Despite the drawbacks of this adaptation, Figure 7 demonstrates successful control of the CH_4_ to O_2_ ratio at the inlet of ref-II, thus, allowing for the avoidance of overheating in this unit. This way, the conversion of CH_4_ remains constant as the GHI fraction increases, and the distribution between reactions described by eqs 4 and 5 results in slightly higher H_2_ production and reduced water formation.

**Figure 7:**
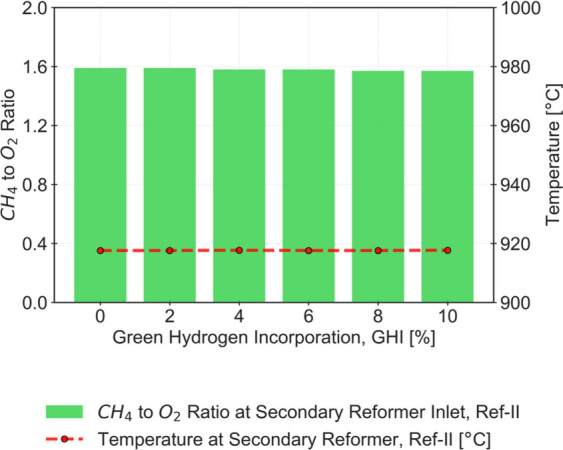
CH_4_ to O_2_ ratio at the ref-II inlet stream (left axis) and outlet ref-II temperature (right axis) for an incorporation of Green H_2_ (GHI) of 0 to 10%, considering an adapted SMR.

To highlight the differences between the two strategies proposed, some metrics for a GHI fraction of 10% are presented in Table 5. The CH_4_ savings documented for Conventional SMR exceed those obtained for Adapted SMR (and even exceed GHI). This phenomenon can be attributed to the overheating in ref-II reported for the first configuration, which marginally increases H_2_ production in this unit, thereby enabling a more significant reduction in natural gas consumption. In contrast, the Adapted SMR promotes a different carbon distribution within the process, resulting in a higher proportion of carbon being released as CO_2_ in outlet streams, which are considered to be the decarbonization metric. As a result, even with lower CH_4_ Savings, the Adapted SMR configuration achieves a higher Decarbonization extent. The carbon flow rates for these scenarios can be found in Table S7 of the Supporting Information.

**Table 5:** Process Metrics between Conventional and Adapted SMR Operation for a GHI of 10%

	Bypass Fraction [%]	CH_4_ Savings [%]	Total Decarbonization [%]	ref-I Reduction Load [%]	ref-II Reduction Load [%]
Conventional SMR	-	10.5	8.1	11.5	7.3
Adapted SMR	21	10.0	9.6	28.3	5.6

This divergence between CH_4_ Savings and Total Decarbonization highlights the need to clearly distinguish the scope and interpretation of the metrics employed. While the CH_4_ Savings metric should be primarily viewed from an economic standpoint (as it measures reductions in natural gas consumption), the Total Decarbonization metric should be analyzed from an environmental viewpoint (as it measures the decrease in carbon emissions released directly to the atmosphere).

With these results, the ref-I bypass demonstrates the technical feasibility of a modified SMR operation. However, further work is necessary to evaluate its economic viability, considering not only green H_2_ production costs but also the potential efficiency losses of the SMR process when deviating from its nominal conditions.

Once the ref-I bypass ensured control of the outlet temperature, the further incorporation of green H_2_ was studied. Figure 8 shows the outlet temperature behavior and evolution of the ref-I bypass fraction for incorporations ranging from 0 to 60%. The results are available in Table S8 of the Supporting Information.

**Figure 8:**
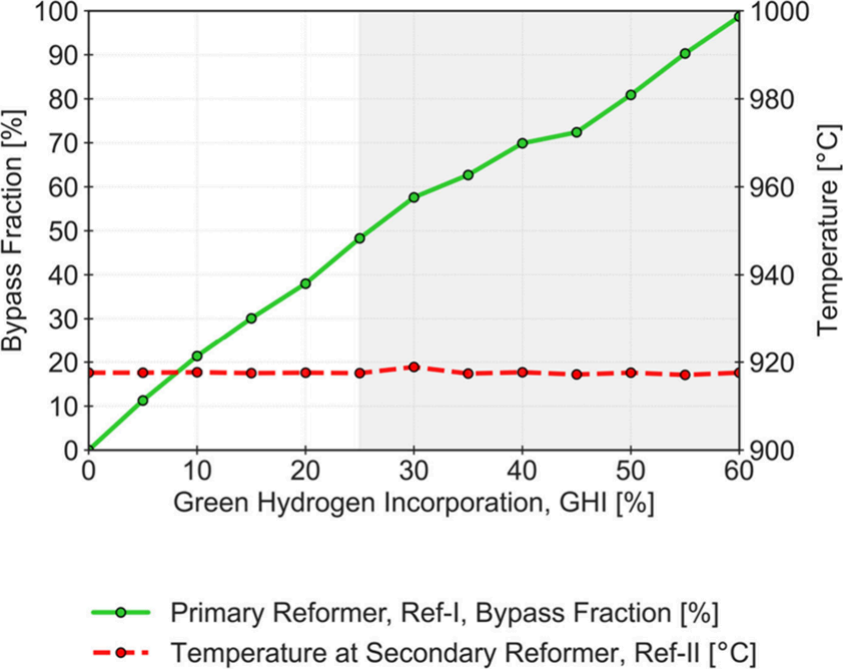
Bypass fraction (left axis) and Secondary Reformer temperature (right axis) for an incorporation of Green H_2_ (GHI) of 0 to 60%, considering an adapted SMR (gray shaded area corresponds to a mass flow rate reduction in ref-I higher than 60%).

Adjusting the bypass fraction enables effective control of temperature in the secondary reformer up to about 60% of GHI. At this point, the ref-I bypass fraction reaches almost 100%, and Total Decarbonization is about 47.8%. The total bypass operation means that ref-I is no longer in operation, and ref-II becomes the sole reforming unit processing natural gas. This point is a critical factor in the present analysis, as the implementation of green H_2_ is constrained by the inability to regulate the temperature of ref-II. Further increases in GHI above 60%, even with a complete ref-I bypass, would lead to the overheating of this unit, showing a behavior similar to what occurs at lower GHI levels without considering ref-I bypass (see Figure 4). While the complete ref-I bypass demonstrates the theoretical flexibility of the system, this scenario should be regarded as mostly academic. In practical applications, exclusive reliance on ref-II decreases the overall H_2_ yield of the reforming process, which, although advantageous for integrating green H_2_, results in economic disadvantages.

However, operational issues related to the Primary Reformer load may occur even if GHI is lower than 60%. The high bypass fractions obtained imply that ref-I is operated with low flow rates, which may require additional operational strategies. To highlight these issues, GHI values higher than 25% are gray shaded in Figure 8, corresponding to cases where ref-I bypass fractions greater than 50% are reported. For these values, compared to the base case (GHI = 0%), the mass flow rate in ref-I consistently decreases by more than 60%. It is important to note that a GHI fraction of around 25% is then considered a more conservative operational threshold, achieving a Total Decarbonization of 23.4%. Even at this reduced level, the results are still substantively meaningful, indicating a notable degree of operational flexibility in incorporating green H_2_ without adversely affecting the process stability.

Compared to Isella et al. 2024, this technical limit of 25% for green H_2_ incorporation must be understood in light of two constraints: the minimum operational load of the Primary Reformer and the complete avoidance of overheating reported in the Secondary Reformer. Isella et al. defined a maximum acceptable overheating of +100 °C for the latter unit and achieved around 22% of green H_2_ incorporation. When the same criteria are applied to this work, the proposed Primary Reformer bypass operation allows the process to reach a GHI of approximately 34%. This demonstrates that, under comparable assumptions, the bypass configuration offers additional operational flexibility and can effectively increase green H_2_ incorporation into conventional NH_3_ plants.

## 4 Conclusions

In this paper, the incorporation of green hydrogen (H_2_) into a conventional ammonia (NH_3_) plant is studied. This incorporation aims to achieve partial decarbonization of the plant and reduce the environmental impact of NH_3_ production. The effects of this incorporation are analyzed, highlighting two crucial factors: the flow reduction through reforming equipment and the overheating of the outlet gas in the Secondary Reformer (ref-II). An increase of 80 °C was reported at the Secondary Reformer outlet for a green H_2_ incorporation fraction (GHI) of 10%. To mitigate these effects and leverage the GHI value, a new approach is presented: an adapted SMR with a Primary Reformer (ref-I) bypass.

Although it offers lower natural gas savings, the bypass approach can be an interesting and low-cost option for lower GHI, due to the resulting sharp reduction in the mass flow rate through Primary Reformer, which limits this strategy for higher incorporations. By operating a total ref-I bypass (rendering this unit nonoperational), a GHI of 60% can be achieved without overheating ref-II. For smaller GHIs, constraints regarding the minimum load capacity of ref-I may limit green H_2_ incorporation to 25%. Considering an overheating of +100 °C in ref-I, it is possible to achieve GHI fractions as high as 34%, which is a significant development with respect to previous work.

The present study demonstrated the technical feasibility of incorporating green H_2_ into the conventional process and defined its operational limits. The findings can serve as a reference for scenarios in which an external H_2_ supply is available in varying fractions. When the externally supplied H_2_ is green (or at least low-carbon), these results are relevant within a transitional pathway aimed at gradually decarbonizing the NH_3_ industry.

Green H_2_ incorporation, along with ref-I bypass operation, still leads to a certain extent of decarbonization in the conventional NH_3_ process, which should be analyzed as a singular plant focused solely on NH_3_ production. Although many existing NH_3_ plants are coupled with urea synthesis (utilizing the CO_2_ from SMR as a feedstock), this dependency is not so crucial for emerging NH_3_ applications that demand substantial decarbonization extents.

By demonstrating the viability of this approach to mitigate the primary effects of integrating green H_2_ into a conventional plant, this paper unlocks the possibility of a more in-depth study of how various strategies can be considered to decarbonize the conventional process, specifically by considering economic factors and legislation targets. In particular, the simultaneous supply of green H_2_ and nitrogen obtained by an Air Separation Unit will be considered to overcome process constraints further and achieve more holistic decarbonization strategies.

## Supplementary Material



## Notes

Process flowsheet, simulation details for relevant unit operations, and stream tables (PDF)

## Notes

The authors would like to dedicate this article to the beloved memory of Professor Pedro Castro and acknowledge the important role he played in the initial development of the key concepts presented in this work. As we are still learning to live without his presence, we will continue to cherish his example as a researcher, teacher, and friend.

## References

[1] Hailes O. (2024). From Guano
to Green Hydrogen: Food Security and Fertilizer Disputes in International
Energy Law. Journal of International Economic
Law.

[2] Smith C., Hill A. K., Torrente-Murciano L. (2020). Current and Future Role of Haber-Bosch
Ammonia in a Carbon-Free Energy Landscape †. Energy Environ. Sci..

[3] Rouwenhorst, K. ; Castellanos, G. International Renewable Energy Agency; Ammonia Energy Association. Innovation Outlook: Renewable Ammonia; 2022.

[4] Our World in Data . World population supported by synthetic nitrogen fertilizers. https://ourworldindata.org/grapher/world-population-supported-by-synthetic-nitrogen-fertilizers (accessed 2025–03–19).

[5] IEA International Energy Agency . Ammonia Technology Roadmap - Towards More Sustainable Nitrogen Fertiliser Production; 2021. https://www.iea.org/reports/ammonia-technology-roadmap (accessed 2024–10–14).

[6] European Comission . Reference Document on Best Available Technique for the Manufacture of Large Vol. Inorganic Chemicals - Ammonia, Acids and Fertilisers; 2007.

[7] MacFarlane D. R., Cherepanov P. V., Choi J., Suryanto B. H. R., Hodgetts R. Y., Bakker J. M., Ferrero Vallana F. M., Simonov A. N. (2020). A Roadmap to the
Ammonia Economy. Joule.

[8] Gezerman A. O. (2022). A Critical
Assessment of Green Ammonia Production and Ammonia Production Technologies. Kemija u industriji.

[9] Narciso D. A. C., Pires J. M., Fortunato J., Teixeira P., Castro P. M., Pinheiro C. I. C., Matos H. A. (2025). Design and Operation of Power-to-Ammonia
Systems: A Review. Energy Convers Manag.

[10] European Parliament ; Council of the European Union. Directive (EU) 2023/2413 of the European Parliament and of the Council of 18 October 2023 Amending Directive (EU) 2018/2001, Regulation (EU) 2018/1999 and Directive 98/70/EC as Regards the Promotion of Energy from Renewable Sources, and Repealing Council Directive (EU) 2015/652. Official Journal of the European Union 2023, No. L 2413.

[11] International PtX Hub . EU Requirements for Renewable Hydrogen and Its Derivatives; Berlin, 2023.

[12] Valera-Medina, A. ; Banares-Alcantara, R. Techno-Economic Challenges of Green Ammonia as an Energy Vector; 2021.

[13] Jiang S. F., Tang Y., Zheng X. Y., Zhao M. (2025). Electrocatalytic Synthesis
of Ammonia Using Transition Metal-Based Catalysts under Ambient Conditions:
A Review. Environmental Chemistry Letters.

[14] Ishaq H., Shehzad M. F., Crawford C. (2023). Transient
Modeling of a Green Ammonia
Production System to Support Sustainable Development. Int. J. Hydrogen Energy.

[15] Wu Y., Zhao T., Tang S., Wang Y., Ma M. (2024). Research on
Design and Multi-Frequency Scheduling Optimization Method for Flexible
Green Ammonia System. Energy Convers Manag.

[16] Florez J., AlAbbad M., Vazquez-Sanchez H., Morales M. G., Sarathy S. M. (2024). Optimizing
Islanded Green Ammonia and Hydrogen Production and Export from Saudi
Arabia. Int. J. Hydrogen Energy.

[17] Yu Z., Lin J., Liu F., Li J., Zhao Y., Song Y., Song Y., Zhang X. (2024). Optimal Sizing
and Pricing of Grid-Connected
Renewable Power to Ammonia Systems Considering the Limited Flexibility
of Ammonia Synthesis. IEEE Transactions on Power
Systems.

[18] Bose A., Lazouski N., Gala M. L., Manthiram K., Mallapragada D. S. (2022). Spatial Variation in Cost of Electricity-Driven Continuous
Ammonia Production in the United States. ACS
Sustain Chem. Eng..

[19] Wang H., Daoutidis P., Zhang Q. (2021). Harnessing the Wind Power of the
Ocean with Green Offshore Ammonia. ACS Sustain
Chem. Eng..

[20] Kong B., Zhang Q., Daoutidis P. (2024). Nonlinear
Model Predictive Control
of Flexible Ammonia Production. Control Eng.
Pract.

[21] Tully Z., King J., Johnson K. (2025). Hydrogen Storage
Minimization under
Industrial Flexibility Constraints: A Techno-Economic Analysis of
off-Grid Green Ammonia Production. Int. J. Hydrogen
Energy.

[22] Zhang X., Li G., Zhou Z., Nie L., Dai Y., Ji X., He G. (2024). How to Achieve Flexible Green Ammonia Production: Insights via Three-Dimensional
Computational Fluid Dynamics Simulation. Ind.
Eng. Chem. Res..

[23] Fahr S., Kender R., Bohn J. P., Rehfeldt S., Peschel A., Klein H. (2025). Dynamic Simulation
of a Highly Load-Flexible Haber-Bosch Plant. Int. J. Hydrogen Energy.

[24] Cheema I. I., Krewer U. (2018). Operating Envelope
of Haber-Bosch Process Design for
Power-to-Ammonia. RSC Adv..

[25] Nayak-Luke R. M., Bañares-Alcántara R. (2020). Techno-Economic
Viability of Islanded
Green Ammonia as a Carbon-Free Energy Vector and as a Substitute for
Conventional Production †. Cite this:
Energy Environ. Sci..

[26] Campion N., Nami H., Swisher P. R., Vang Hendriksen P., Münster M. (2023). Techno-Economic Assessment of Green
Ammonia Production
with Different Wind and Solar Potentials. Renewable
and Sustainable Energy Reviews.

[27] Fertiberia . H2F Project - Fertiberia. https://www.fertiberia.com/en/greenammonia/h2f-project/ (accessed 2025–03–19).

[28] Musa T., Mahmoud N., Challiwala M. S., Mohamed E., Choudhury H., Elbashir N. O. (2026). Decarbonizing Ammonia Synthesis Plants through Retrofitting
Novel Reformer Technology. Scientific Reports
2025.

[29] Pan L., Li J., Huang J., An Q., Lin J., Mujeeb A., Xu Y., Li G., Zhou M., Wang J. (2023). Renewable-to-Ammonia:
Configuration Strategy and Technoeconomic Analysis. iScience.

[30] Isella A., Ostuni R., Manca D. (2024). Towards the Decarbonization
of Ammonia
Synthesis - A Techno-Economic Assessment of Hybrid-Green Process Alternatives. Chemical Engineering Journal.

[31] Appl, M. Ammonia, 2. Production Processes. In Ullmann’s Encyclopedia of Industrial Chemistry; Wiley, 2011. 10.1002/14356007.o02_o11.

[32] Rase, H. F. Handbook of Commercial Catalysts: Heterogeneous Catalysts; CRC Press, 2000.

[33] Hyde, C. Low US natgas prices help ammonia economics. Argus Media Group. https://www.argusmedia.com/pt/news-and-insights/latest-market-news/2566315-low-us-natgas-prices-help-ammonia-economics (accessed 2025–07–14).

