# Supplementary material for: Toward the Decarbonization of Ammonia Production through the Gradual Incorporation of Green Hydrogen
Source: Ind Eng Chem Res. 2026 Feb 24;65(9):5035–45. doi: 10.1021/acs.iecr.5c03851 (PMC12985266; doi:10.1021/acs.iecr.5c03851)
Supplement: Supplementary file 1 [file ie5c03851_si_001.pdf]

Supporting Information

Towards the Decarbonization of Ammonia Production through the Gradual Incorporation of Green Hydrogen

João Fortunato, Diogo A. C. Narciso, Henrique A. Matos*

Centro de Recursos Naturais e Ambiente, Departamento de Engenharia Química, Instituto Superior Técnico, Universidade de Lisboa, Av. Rovisco Pais 1, 1049-001, Lisboa, Portugal

Corresponding author e-mail: diogo.narciso@tecnico.ulisboa.pt

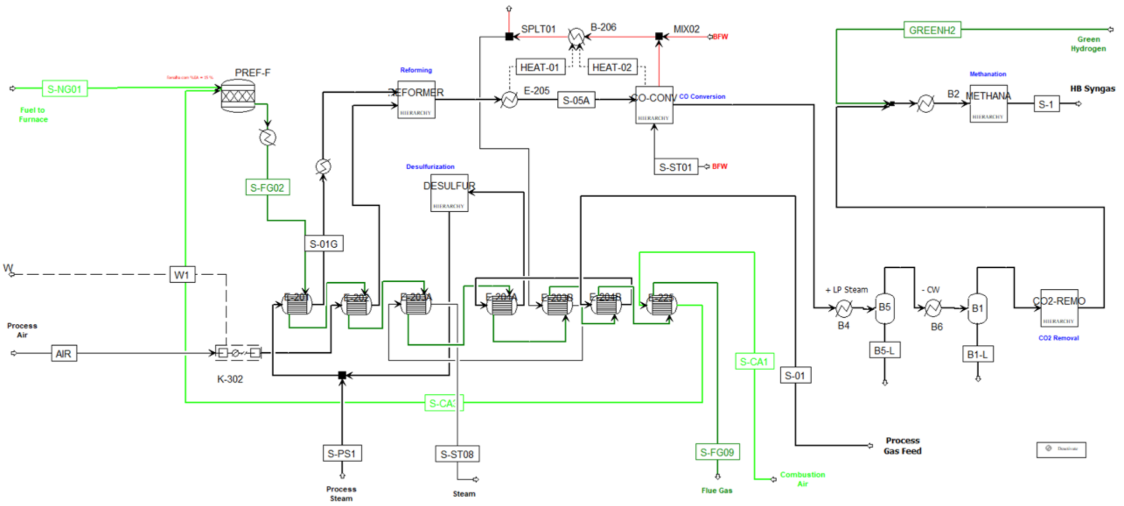

Figure S1. Global flowsheet of the Steam Methane Reforming (SMR) section, simulated in Aspen Plus® V14 including Desulphurization, Reforming, CO Shift, CO_2 Removal and Methanation subsections.

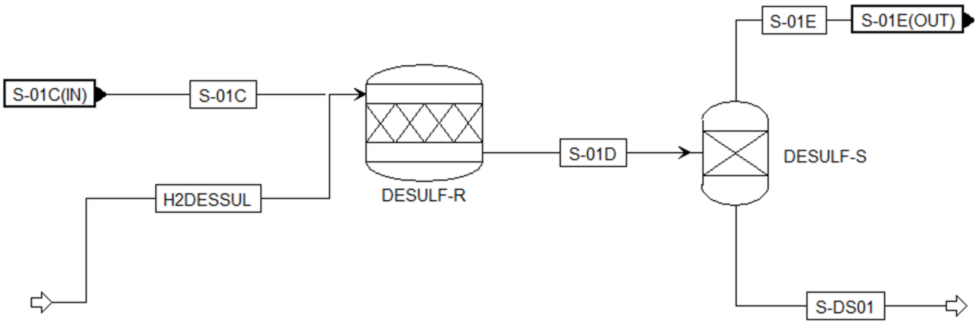

Figure S2. Flowsheet of the Desulphurization section, simulated in Aspen Plus® V14.

Table S1. Relevant information about the main units of the Desulphurization section.

Unit	Block Type	Relevant Information
DESULF-R	RStoic	Pressure = 35 bar<br>Duty = 0 MMkcal/hr
DESULF-S	Sep2	Split Fraction (H_2S) = 1

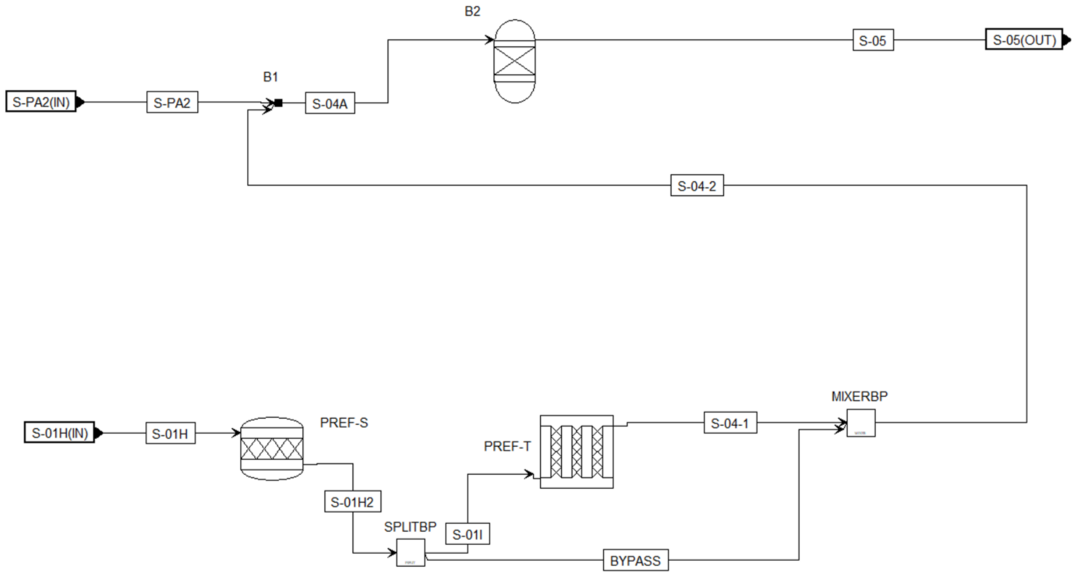

Figure S3. Flowsheet of the Reforming section, simulated in Aspen Plus® V14.

Table S2. Relevant information about the main units of the Reforming section.

Unit	Block Type	Relevant Information
PREF-S	RStoic	Pressure Drop = 1.9 bar<br>Duty = 0 MMkcal/hr<br>Frac. Conversion (Heavy Hydrocarbons) = 1
PREF-T	RPlug	Pressure Drop = 2 bar<br>Number of Tubes = 82<br>Tube Length = 10 m<br>Tube Diameter = 0.14 m
B2	RGibbs	Duty = 0 MMkcal/hr<br>Inerts = N_2, Ar

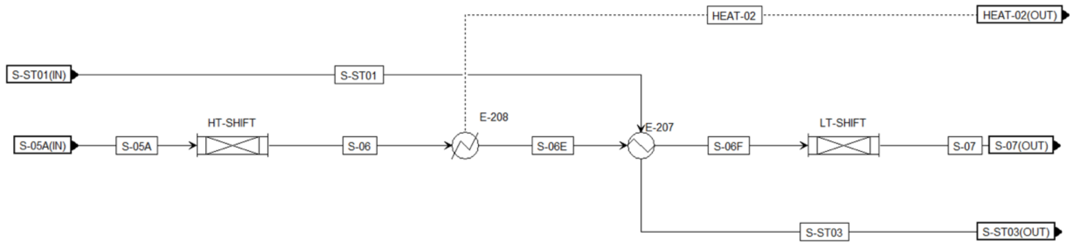

Figure S4. Flowsheet of the CO Shift section, simulated in Aspen Plus® V14.

Table S3. Relevant information about the main units of the CO Shift section.

Unit	Block Type	Relevant Information
HT-SHIFT	RPlug	Adiabatic Reactor<br>Length = 15.8 m<br>Diameter = 2.2 m
LT-SHIFT	RPlug	Adiabatic Reactor<br>Length = 7.7 m<br>Diameter = 3.7 m

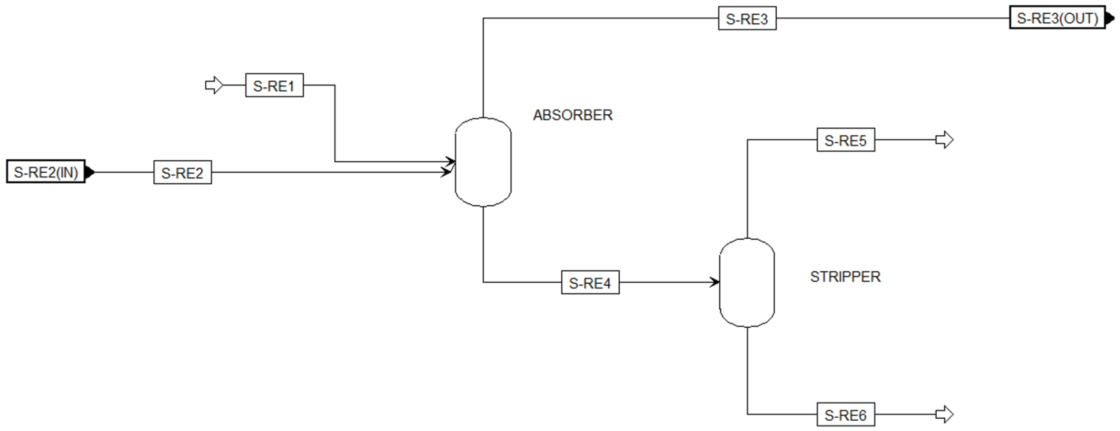

Figure S5. Flowsheet of the CO_2 Removal section, simulated in Aspen Plus® V14.

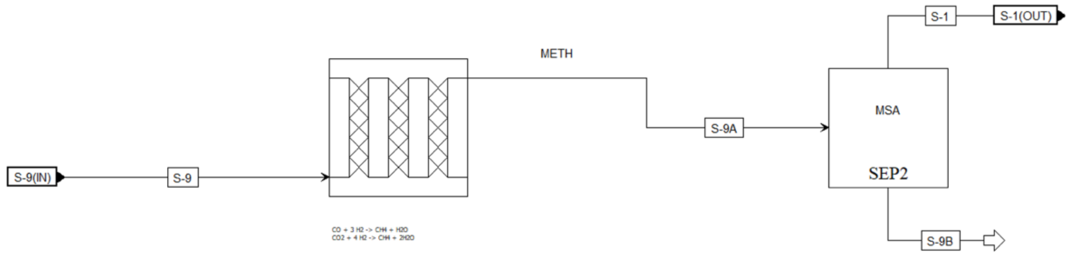

Figure S6. Flowsheet of the Methanation section, simulated in Aspen Plus® V14.

Table S4. Relevant information about the main units of Methanation section.

Unit	Block Type	Relevant Information
METH	RPlug	Adiabatic Reactor<br>Number of Tubes = 30<br>Tube Length = 3 m<br>Tube Diameter = 0.11 m
MSA	Sep2	Split Fraction (H_2O) = 1<br>Split Fraction (H_2) = 0.017<br>Split Fraction (N_2, Ar, CH_4) = 0.01

The *Aspen Plus V14* Optimization Tool uses **Sequential Quadratic Programming (SQP)** (more information can be found in *Aspen Plus User Guide*, available at https://web.ist.utl.pt/ist11038/acad/Aspen/AspUserGuide10.pdf) and provided the following results for each fraction of Green Hydrogen Incorporation (GHI).

Table S5. Results obtained for a Green Hydrogen Incorporation of 0 to 10 %.

**Green H_2 [%]**		**0**	**2**	**4**	**6**	**8**	**10**
1.Process Natural Gas	[ton/hr]	16,2	15,8	15,4	15,0	14,7	14,4
2.Process Steam	[ton/hr]	51,1	49,7	48,5	47,4	46,3	45,3
3.Process Air	[ton/hr]	39,2	39,2	39,2	39,2	39,2	39,2
CO_2 Removal Solution	[ton/hr]	133,9	131,7	129,5	127,2	125,0	122,8
A.Fuel Natural Gas	[ton/hr]	4,7	4,6	4,5	4,4	4,4	4,3
B.Combustion Air	[ton/hr]	93,1	92,1	90,0	88,8	87,6	86,5
Ref-II Outlet Stream Temperature	[ºC]	918	932	943	957	971	986

Table S6. Results obtained for a Green Hydrogen Incorporation of 0 to 10 % (adapted SMR).

**Green H_2 [%]**		**0**	**2**	**4**	**6**	**8**	**10**
1.Process Natural Gas	[ton/hr]	16,2	15,9	15,6	15,4	15,1	14,8
2.Process Steam	[ton/hr]	51,1	50,2	49,3	48,4	47,5	46,6
3.Process Air	[ton/hr]	39,2	39,2	39,2	39,2	39,2	39,2
CO_2 Removal Solution	[ton/hr]	133,9	131,6	129,5	127,2	125,0	122,9
A.Fuel Natural Gas	[ton/hr]	4,7	4,5	4,4	4,3	4,1	4,0
B.Combustion Air	[ton/hr]	93,1	90,1	88,1	85,1	82,6	80,1
Ref-II Outlet Stream Temperature	[ºC]	918	918	918	918	918	918
Ref-I Bypass Fraction	-	0,0	4,4	9,1	13,5	17,8	21,4

Table S7. Carbon flow rates in and out of the SMR section (for a GHI = 0 % and GHI = 6 %, with and without Reformer-I Bypass operation).

		**GHI = 0 % [kmol/hr]**	**GHI = 6 % [kmol/hr]**	**GHI = 6 % [kmol/hr]**
		-	w/out Ref-I BP	w/ Ref-I BP
IN	CH_4 in Process Natural Gas Feed	679.7	629.7	643.6
	Heavy Hydrocarbons in Process Natural Gas Feed	337.8	312.9	319.8
	Process Air Feed	0.4	0.4	0.4
	Total Process Carbon	1017.9	943.0	963.8
	CH_4 in Fuel Natural Gas Feed	195.1	186.2	178.4
	Heavy Hydrocarbons in Fuel Natural Gas Feed	97.0	92.5	88.7
	Combustion Air	1.0	0.9	0.9
	Total Fuel Carbon	293.1	279.6	268.0
	Total Carbon	1311.0	1222.6	1231.8
OUT	Hot and Cold Condensates	-0.3	-0.3	-0.3
	CO_2 Removal Section	-964.6	-908.5	-915.7
	Methanation Section Removal	-0.5	-0.3	-0.5
	HB Synthesis Section Feed	-52.5	-33.9	-47.3
	Total Process Carbon	-1017.9	-943.0	-963.8
	CO_2 Flue Gas	-293.1	-279.6	-267.9
	Total	-1311,0	-1222.6	-1231.7

In the Steam Methane Reforming process, carbon exits the system as CO_2, CH_4, and CO during the CO_2-removal stage, either in the syngas stream sent to the Haber–Bosch synthesis, retained in the final water-adsorption stage, or carried away in condensate streams. The most significant carbon outputs are CO_2 removal (accounting for more than 90 % of the carbon removed, as CO_2) and the syngas stream (around 5 % of the carbon removed, as CH_4).
Although the savings of CH_4 burned as fuel (stream 10. Natural Gas) have an equivalent influence on CO_2 emissions accounted in the respective flue gas (stream 12. Flue Gas), this behavior is not reported in the process line with the consumption of Process CH_4 (stream 1. Process Natural Gas).

Table S8. Results obtained for a Green Hydrogen Incorporation of 0 to 60 % (adapted SMR).

**Green H_2 [%]**		**0**	**5**	**10**	**15**	**20**	**25**	**30**
Ref-II Outlet Stream Temperature	[ºC]	918	918	918	918	918	918	919
Ref-I Bypass Fraction	-	0,0	11,3	21,4	30,1	38,0	48,3	57,6

**Green H_2 [%]**		**35**	**40**	**45**	**50**	**55**	**60**
Ref-II Outlet Stream Temperature	[ºC]	917	918	917	918	917	918
Ref-I Bypass Fraction	-	62,7	69,9	72,4	80,9	90,3	98,7
